# Are optical trocars free of complications?

**DOI:** 10.1002/ccr3.2091

**Published:** 2019-03-09

**Authors:** Roberto Bustos, Mario Masrur, Patrice Frederick

**Affiliations:** ^1^ Division of General, Minimally Invasive and Robotic Surgery, Department of Surgery University of Illinois at Chicago Chicago Illinois

**Keywords:** entry, laparoscopic, optical, port, trocar

## Abstract

Optical view trocars provide an acceptable level of safety for port entry during minimally invasive surgery. Nevertheless, this technique is not exempt from potential complications. These can range from a minor subcutaneous insufflation that will not require any further treatment to abdominal aorta injuries.

A 52‐year‐old male presented with uncomplicated right inguinal hernia. A robot‐assisted transabdominal preperitoneal repair was planned. The patient had no past medical or surgical history besides the hernia. BMI is 28.72 kg/m^2^. A 5 mm optical port was used in the supraumbilical area to gain entry into the abdomen (Video S1). After the cannula was removed, an exploration was attempted, but proper insufflation of the cavity was not achieved and it was not possible to identify any structures.^1^ Decision was made to remove the trocar and repeat the procedure, accomplishing a proper insufflation and access to the peritoneal cavity.

## CONFLICT OF INTEREST

None declared.

## AUTHOR CONTRIBUTION

RB: drafted the article, collected the data and images, and edited the video. MM: critically revised the video and manuscript. PF: designed the work, critically revised and approved the final version.

## Supporting information

Video
